# Impact of superparamagnetic iron oxide nanoparticles on in vitro and in vivo radiosensitisation of cancer cells

**DOI:** 10.1186/s13014-021-01829-y

**Published:** 2021-06-12

**Authors:** Emily Russell, Victoria Dunne, Ben Russell, Hibaaq Mohamud, Mihaela Ghita, Stephen J. McMahon, Karl T. Butterworth, Giuseppe Schettino, Conor K. McGarry, Kevin M. Prise

**Affiliations:** 1 Patrick G. Johnston Centre for Cancer Research, Queen’s University, Belfast, UK; 2 National Physical Laboratory, London, UK; 3 Department of Medical Physics and Engineering, Leeds Teaching Hospitals, NHS Trust, Leeds, UK; 4 Department of Physics, University of Surrey, Guildford, UK; 5 Northern Ireland Cancer Centre, Belfast, UK

**Keywords:** Nanoparticles, Radiobiology

## Abstract

**Purpose:**

The recent implementation of MR-Linacs has highlighted theranostic opportunities of contrast agents in both imaging and radiotherapy. There is a lack of data exploring the potential of superparamagnetic iron oxide nanoparticles (SPIONs) as radiosensitisers. Through preclinical 225 kVp exposures, this study aimed to characterise the uptake and radiobiological effects of SPIONs in tumour cell models in vitro and to provide proof-of-principle application in a xenograft tumour model.

**Methods:**

SPIONs were also characterised to determine their hydrodynamic radius using dynamic light scattering and uptake was measured using ICP-MS in 6 cancer cell lines; H460, MiaPaCa2, DU145, MCF7, U87 and HEPG2. The impact of SPIONs on radiobiological response was determined by measuring DNA damage using 53BP1 immunofluorescence and cell survival. Sensitisation Enhancement Ratios (SERs) were compared with the predicted Dose Enhancement Ratios (DEFs) based on physical absorption estimations. In vivo efficacy was demonstrated using a subcutaneous H460 xenograft tumour model in SCID mice by following intra-tumoural injection of SPIONs.

**Results:**

The hydrodynamic radius was found to be between 110 and 130 nm, with evidence of being monodisperse in nature. SPIONs significantly increased DNA damage in all cell lines with the exception of U87 cells at a dose of 1 Gy, 1 h post-irradiation. Levels of DNA damage correlated with the cell survival, in which all cell lines except U87 cells showed an increased sensitivity (*P* < 0.05) in the linear quadratic curve fit for 1 h exposure to 23.5 μg/ml SPIONs. There was also a 30.1% increase in the number of DNA damage foci found for HEPG2 cells at 2 Gy. No strong correlation was found between SPION uptake and DNA damage at any dose, yet the biological consequences of SPIONs on radiosensitisation were found to be much greater, with SERs up to 1.28 ± 0.03, compared with predicted physical dose enhancement levels of 1.0001. In vivo, intra-tumoural injection of SPIONs combined with radiation showed significant tumour growth delay compared to animals treated with radiation or SPIONs alone (*P* < 0.05).

**Conclusions:**

SPIONs showed radiosensitising effects in 5 out of 6 cancer cell lines. No correlation was found between the cell-specific uptake of SPIONs into the cells and DNA damage levels. The in vivo study found a significant decrease in the tumour growth rate.

**Supplementary Information:**

The online version contains supplementary material available at 10.1186/s13014-021-01829-y.

## Introduction

Over the last few decades, radiotherapy has become increasingly conformal through improvements in treatment planning and the introduction of new delivery techniques. This accurate dose delivery has led to a growth in research into how to further protect healthy tissues, including the use of hypofractionated treatments [1, 2] and high dose rate FLASH radiotherapy treatments [3–5]. However, research is also discovering ways to enable greater tumour radiosensitisation through drug radiotherapy combination approaches [6], including the use of metal-based nanoparticles [7].

Nanoparticles (NPs), particularly high Z number metal elements, have been extensively studied due to their high absorption of X-ray photons leading to large increases in secondary electrons and subsequent increase levels of DNA damage by physically enhancing the dose [8–10]. However, high Z metal NPs have also been shown to act through distinct biological mechanisms including higher levels of oxidative stress, increasing levels of reactive oxygen species (ROS) and inducing highly reactive hydroxyl radicals which go on to cause further DNA damage [10–17].

High Z number metal NPs have significant potential in radiotherapy as they are preferentially taken up by the tumour, ensuring that there is no increase in DNA damage to healthy surrounding tissue. This is due to the enhanced permeability and retention (EPR) effect, by which the leaky vasculature of the tumour takes up the NPs, where the size of the particles would otherwise be too large for healthy cells with normal vasculature [18–20]. As NPs are normally larger than the renal excretion threshold, they are also likely to stay in the tumour for a longer period, potentially causing greater levels of radiosensitisation [21].

The focus of much of the research into nanoparticle radiosensitisation has involved gold nanoparticles. They have proved to be effective in a range of sizes, with various coatings and chelating agents [22–34]. However, more recently, gadolinium nanoparticles have been studied [35–45], with AGuIX (a 5 nm gadolinium nanoparticle) currently being tested in the NanoRAD2 clinical trial [46]. The optimal element for NP radiosensitisers is under debate, with McMahon et al*.* demonstrating both the micro- and macroscopic factors for a range of elements to determine the optimal nanoparticle element[47].

Superparamagnetic Iron Oxide Nanoparticles (SPIONs) have been studied for a range of different treatments, from drug delivery to antibody-binding [48], however, they are primarily investigated for their ability to provide magnetic hyperthermia treatments [49–52]. The superparamagnetic properties of SPIONs mean that, when placed in an alternating magnetic field, they vibrate and heat up, but once removed from the magnetic field, this magnetism is lost [50]. When placed inside tissues, it is thought that this hyperthermia effect induces cell death [48].

The rationale for selecting SPIONs in this study is due to its applicability to the ongoing development of MR-Linacs. Iron oxide contrast agents have been used primarily in T2-weighted MRI scans [48, 53], and so, with the combination of MRI and radiotherapy now becoming clinically available, it is possible that these MRI contrast agents could be used both diagnostically and therapeutically, both for contrast enhancement but also for increasing the radiosensitivity of a tumour for more effective radiotherapy treatments. A study by Hu et al. demonstrated this capability with the gadolinium-based nanoparticle, AGuIX [54].

Some previous studies have evaluated the radiosensitising properties of SPIONs, including Klein et al. indicating radiosensitisation with MCF7 breast cells through cytotoxicity studies and production of reactive oxygen species [55]. Kirakli et al. also investigated the radiosensitisation of citrate coated SPIONs using MV radiation on 3 cancer cell lines using clonogenic assays, finding the greatest radiosensitisation at a dose of 2 Gy [56]. Rashid et al. investigated radiosensitisation of colon cancer cells from SPIONs in combination with a 150 MeV proton beam, investigating the reactive oxygen species generation [13]. The use of iron oxide nanoparticles in combination with proton beams have also been investigated in vivo by Seo et al. [57]. Choi et al. found 13 nm iron oxide nanoparticles to significantly reduce the viability of colon tumour cells in mice compared with control cells when treated with 7.1 keV monochromatic x-rays [58].

There is a clinical need to determine the efficacy of SPIONs for the development of MR-Linacs with potential capabilities of utilising them both diagnostically and therapeutically, which should be determined using further studies. The aim of this study is to investigate the impact of SPIONs on the radiosensitisation in vitro when combined with 225 kVp X-rays through clonogenic and DNA damage immunofluorescence assays. The SPIONs’ hydrodynamic radius was characterised using Dynamic Light Scattering (DLS). Inductively-Coupled Plasma Mass-Spectrometry (ICP-MS) has also been used to quantify the iron oxide taken into cells, before correlating with the levels of DNA damage. This radiosensitisation was then investigated in vivo for H460 subcutaneous tumours.

It is also important to note the difference between the kVp source used for this investigation and the MV energies which are conventionally used for radiotherapy. kVp energies are dominated by the photoelectric effect which is proportional to Z^3^ compared with the Compton effect for MV energies which is proportional to Z [59], suggesting that radiosensitisation from the nanoparticles would be greater for kVp energies. However, the largest differences in the ratios of absorption coefficients between soft tissue and nanoparticles occur in the region below 100 keV [60] which are strongly attenuated by the 2 mm Cu filter used for the kVp beams (Additional file 2). In particular, the k-edge of iron oxide is < 10 keV, further reducing the effect [60]. Whilst it is important to acknowledge the differences in the physical dose absorption caused by the differences in energy, comparison can be made regarding the extent of the biological effects.

## Materials and methods

### Cell culture and nanoparticles

6 human cancer cell lines were selected to be investigated in vitro; H460 (large cell lung cancer), MiaPaCa2 (pancreatic carcinoma), DU145 (prostate carcinoma), MCF7 (breast adenocarcinoma), U87 (brain glioblastoma) and HEPG2 (liver carcinoma). All cell lines were acquired from the American Type Culture Collection (ATCC), and were routinely tested for mycoplasma. All cell lines have also been authenticated by ATCC using Short Tandem Repeat (STR) Screening. Cells were incubated at 37 °C in 5% CO_2_, and all tissue culture was performed in a Class II laminar flow cabinet (Thermo, US). All cell lines, except for MiaPaCa2, were cultured in Dulbecco’s Modified Eagle Media (DMEM) (Sigma, US), supplemented with 10% Foetal Bovine Serum (FBS) (Sigma, US) and 1% Penicillin Streptomycin (Pen-Strep) (Sigma, US). MiaPaCa2 cells were incubated in High Glucose DMEM media, supplemented with 10% FBS, 1% Pen-Strep, and 0.2% Sodium Pyruvate. Cells were passaged every 2–3 days to maintain exponential grown.

NPs used for this study were SPIONs with a 5 nm diameter, acquired from Sigma, US (product number 725331), suspended in H_2_O at a concentration of 5 mg/ml, with the molecular weight of Fe_3_O_4_ being 231.53 g/mol. For the purpose of this study, all assays were performed at a SPION concentration of 23.5 μg/ml, which was determined as being within the range investigated by Kirakli et al. [56].

### Dynamic light scattering

The SPIONs used for this study are commercially available and quoted as being 5 nm in diameter, determined by Transmission Electron Microscopy (TEM) [61]. However, the size of the molecules when dispersed in an aqueous solution is unclear. DLS was used to quantify the diameter of hydrodynamic radius and provide detail about the nature of the dispersion.

The experiment was carried out at the National Physical Laboratory, and followed the methods described by Minelli et al. [62]. This is a validated method for determining nanoparticle size. A Zetasizer (Zetasizer Nano ZS 3600, Malvern Panalytical Ltd, Malvern, UK) was used which performs measurements using a 633 nm Helium-Neon laser, measuring the light at a scattered angle of 173°. The particles were diluted to a range of concentrations; stock dilution, 1:1, 1:4, 1:6 and 1:20, with measurements carried out at temperature of 25°C and 3 repeats for each sample. The average diameter for each sample was noted along with peaks in the measurements to determine any variation in size of agglomeration of the particles.

### ICP-MS uptake measurements

ICP-MS was used to determine the quantity of the SPIONs that were entering the cells. For preparation of ICP-MS samples, 1 × 10^6^ cells were plated into 30 mm petri-dishes, before adding SPIONs at a concentration of 23.5 μg/ml in 500 μl media, 24 h later. After a further 24 h, in order to adhere to the incubation times used for in vitro experiments, the cells were washed with phosphate buffered solutions (PBS) (Sigma, US), suspended using 2 ml trypsin (Sigma, US), and the number of cells counted using a Coulter Counter (Beckman Coulter Life Sciences, US). Cells were covered in 500 μl deionised water (ddH_2_O) and left to dry overnight. Cells were then exposed to 500 μl of Aqua Regia (3: 1 hydrochloric acid: nitric acid) and left in a fume hood overnight to evaporate, to dissolve the cells so that only the NPs remained. The remaining SPIONs were resuspended into 30 ml of ddH_2_O prior to running the samples.

The ICP-MS (Agilent 8800, Agilent Technologies, UK) ran the samples consecutively using an autosampler, with a regular sample blank to test for any iron contamination. Also, one SPION sample was sampled repeatedly as an internal standard over the course of the run to test for evidence of SPIONs falling out of solution. Any decrease in detected signal found was then corrected. The ICP-MS machine is equipped with two quadrupole mass filters and a collision-reaction cell, but in this study was operated with only one mass filter (termed single quad mode). Helium was introduced into the cell at a flow rate of 4 ml/min as a collision gas to remove ^40^Ar^16^O formed in the plasma, which would otherwise overlap with the ^56^Fe signal. The instrument was fitted with a quartz double-pass spray chamber as well as a MicroMist nebuliser (Glass Expansion, Australia), with nickel sample and skimmer cones (Crawford Scientific, UK). This setup was in accordance to Russell et al. [63]. Between each of the samples the probe was rinsed twice, firstly with 0.3 M HNO_3_ to wash Fe from the previous sample and prevent any cross contamination between samples, and then with ddH_2_O to match the matrix of the next sample to be measured.

Using a calibration curve made from a range of samples with known concentrations of SPIONs, the counts per second detected by the ICP-MS were converted to a corresponding concentration to determine the mass of iron oxide found on average within each cell.

### Immunofluorescence DNA damage assay

DNA double strand breaks (DSBs) were detected using a 53BP1 immunofluorescence assay. Cells were seeded onto sterilised cover slips in 6-well plates (Starstedt, Germany), at a cell density of 1 × 10^5^ cells per well, covered with 2 ml media. 24 h later, SPIONs were added at a concentration of 23.5 μg/ml in 500 μl. After a further 24 h, cells were irradiated with doses of 0–2 Gy, and immediately afterwards the nanoparticles were replaced with fresh media. Only doses up to 2 Gy were used because higher doses would result in a high number of overlapping foci leading to inaccurate determinations of the level of DNA damage within the cell. All irradiations were carried out using an X-RAD225 X-ray cabinet source (Precision X-Ray, US). Irradiations were performed at an SSD of 50 cm, with a dose rate of 0.589 Gy/min and an energy of 225 kVp filtered with a copper filter of 2 mm Cu that gives a half value layer (HVL) of 2.3 mm Cu. The mean energy of the x-ray spectrum is 113 kV, with a peak energy occurring around 100 kV.

Cells were fixed at time points of 1 h and 24 h post-irradiation, adding 2 ml of 50: 50 methanol: acetone to each well for an incubation time of 20 min at 4 °C before cells were washed 3 times for 5 min in PBS. Cells were permeabilised using 1 ml methanol per well for 10 min before being washed again 3 times with PBS. Blocking was performed using 2 ml blocking buffer (5% FBS, 0.5% Triton X-100 in PBS), incubated for 1 h at 4 °C. An anti-53BP1 antibody derived from rabbits was used as the primary antibody, at a concentration of 1:3000 suspended in blocking buffer, with 500 μl added per well. After an incubation time of 1 h at room temperature, the primary antibody was washed off 3 times in washing buffer (0.1% Triton X-100 in PBS) for 5 min. The secondary antibody, goat-anti-rabbit, was used (GAR 488) at a concentration of 1:2000 suspended in blocking buffer, at room temperature for 1 h, in darkness to not fade the fluorescence. Samples were washed in washing buffer 3 times and mounted onto glass slides using 20 μl ProLong Gold Antifade Mountant with DAPI (Thermo, US).

To quantify levels of DSBs, the number of foci was counted for 50 randomly selected cells for each slide and averaged over 3 repeats. Statistics were carried out as a Student’s *t* test for any significant increase in DNA damage. When counting foci, cells were discounted if they were enlarged, had very large numbers of foci, or appeared to be undergoing mitosis or apoptosis.

DNA damage was further analysed by comparing with the uptake data using ICP-MS. A correlation was determined between the numbers of foci and the mass of iron oxide taken up using the Spearman’s Rank correlation coefficient, used for non-parametric data, with a coefficient between + / − 1 with 1 being an entirely positive correlation and − 1 being entirely negative. A correction of greater than + / − 0.7 is considered to be a strong correlation [64].

### Clonogenic assay

Cell survival was quantified using the clonogenic assay for a range of doses from 0 to 8 Gy for the combination of radiation combined with SPIONs, using the method described by Puck and Marcus [65]. Firstly, cells were seeded into 6 well plates with cell densities depending on the dose of radiation received; 0–2 Gy: 500 cells, 4 Gy: 1000 cells, and 8 Gy: 2000 cells. Cells were incubated for 24 h to adhere to the wells, before SPIONs were added both 1 h and 24 h prior to radiation at a concentration of 23.5 μg/ml in 500 μl media. After irradiation with 225 kVp X-rays, the SPIONs were replacing with fresh media. They were left for an incubation time ranging from 6 to 13 days, depending on the cell line. Cells were stained using Crystal Violet (0.4% in 95% Ethanol), for 20 min before the excess was rinsed off.

The number of colonies was then counted for each well, with a colony determined to be a group of > 50 cells. The plating efficiency (PE) was calculated to be the percentage of the cells plated that went on to develop colonies. This was used to determine the surviving fraction (SF) for each dose, as the proportion of colonies counted compared with controls. Graphs of radiation dose against surviving fraction were then fitted with the Linear Quadratic Model, given by Eq. 1, where D is the radiation dose (Gy) and $\alpha$ and $\beta$ are the linear and quadradic components of cell killing.

1 $$SF = {\text{exp}}\left( { - \alpha D - \beta D^{2} } \right)$$

Statistical significance in the clonogenic assays was carried out using the Extra Sum-of-Squares F-Test using GraphPad™ Prism, by which it is determined whether one curve of best fit could accurately fit the other data sets.

### Predicted dose enhancement factor (DEF) and sensitisation enhancement ratio (SER)

The SER was calculated using the clonogenic assay and is a ratio of the dose of radiation in combination with SPIONs that gives the same level of cell survival for a specific radiation dose only [66]. This was carried out both for the 8 Gy dose and for a 10% surviving fraction for controls, in which the $\alpha$ and $\beta$ values for the control and the treated linear quadratic curves were used to determine the dose that would give the same surviving fraction for treated cells. An SER of 1 would represent the same biological effect as the control samples.

To predict the DEF, prior to performing the clonogenic assay, it was approximated as the ratio of mass energy absorption coefficients between the media alone and the media plus iron oxide, using the method described by McMahon et al. for macroscopic dose enhancement calculations [47]. Firstly, the attenuation coefficients were determined for a range of energies from 0 to 225 keV, and then a scaling factor accounted for the fluence of the X-Ray spectrum at each energy for both water and iron oxide. The density of the SPIONs in the media was then used to predict the DEF.

### In vivo model

Severe Combined Immunodeficient (SCID) mice at 8–10 weeks old were obtained from Charles River Laboratories (Oxford, UK), 1 × 10^6^ H460 cells were subcutaneously injected into the dorsum of each mouse. Animals were randomly assigned to the following experimental groups; control (n = 5), SPIONs only (n = 2), Radiation only (n = 4), and combined radiation and SPIONs (n = 5). 23.5 μg/ml SPIONs in 50 μl sterile ddH_2_O were intra-tumourally injected 15 min prior to irradiations of 12 Gy in a single fraction with X-ray energy 220 kVp using a Small Animal Radiation Research Platform (SAARP) (Xstrahl, UK), with Cone Beam CT (CBCT) image-guided and parallel opposed beam geometry. Following treatment, tumour volume was measured every 2–3 days using callipers until experimental endpoints were reached which were defined as a tumour geometric mean diameter (GMD) of 12 mm^3^ or a 10% loss in body weight, measured by taking the diameter of the tumour in 3 planes. All procedures were carried out in accordance with United Kingdom Department of Health approval for in vivo experimentations.

## Results

### Dynamic light scattering

**Fig. 1:**
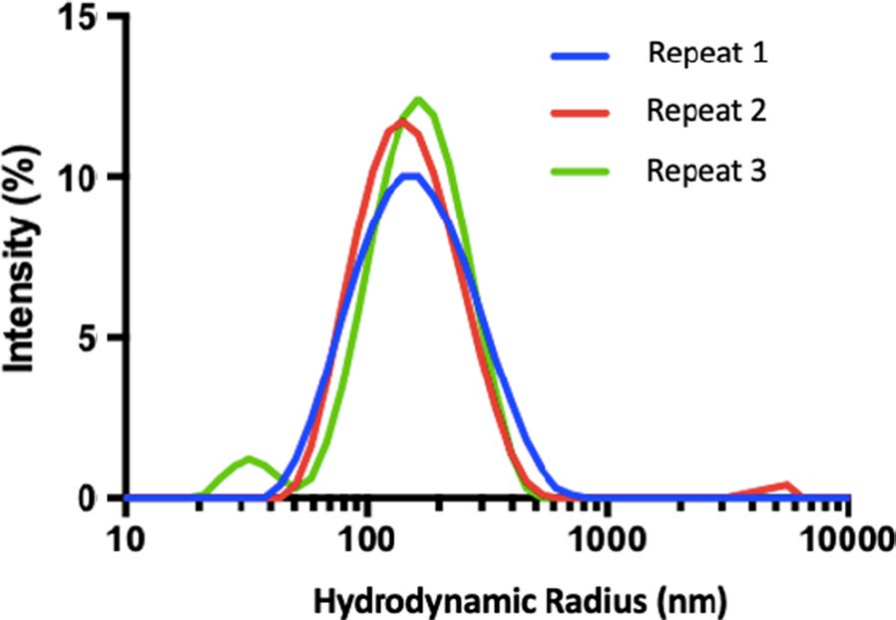
DLS measurements indicating distribution of hydrodynamic radius of SPIONs, at the stock concentration which is the concentration that the supplier provides; 5 mg/ml

The average hydrodynamic radius of the SPIONs was found to be 124 ± 6 nm across all dilutions. This accounts for the size of the nanoparticle plus any shell that forms around the nanoparticles when dispersed in aqueous solution and it may represent a more informative parameter for the cellular uptake. It will also account for anything attached to the nanoparticle including possible nanoparticle aggregation. A graph of diameter against intensity of signal is shown in Fig. 1, indicating a dominant peak around 160 nm and a second smaller peak around 30 nm for 3 repeats at the stock concentration. The largest peak accounts for 98% of the measurements compared with 2% for the smaller peak, suggesting that the SPIONs are predominantly monodisperse. Even at the highest 1:20 dilution, the smaller peak accounted for only 5% of the measurements, suggesting the SPIONs do not agglomerate.

### ICP-MS uptake measurements

**Fig. 2:**
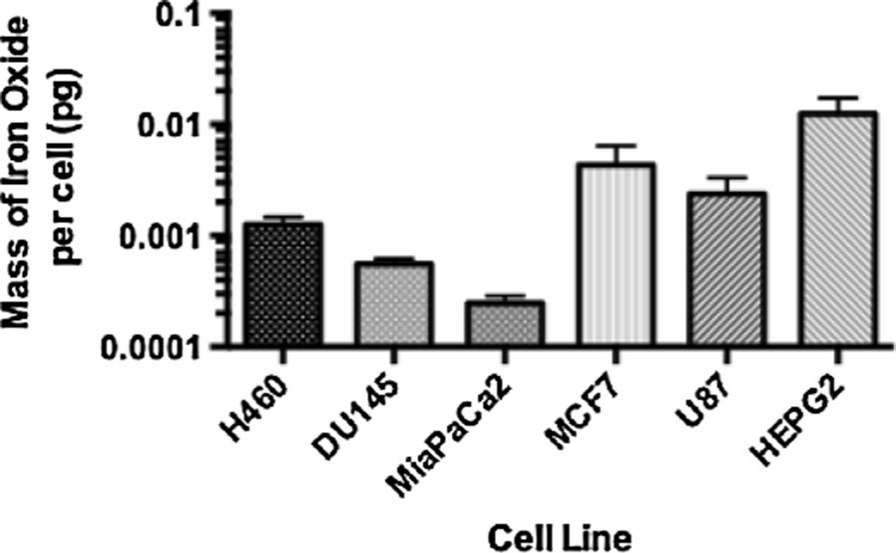
Uptake measurements performed using ICP-MS, representing the mass of iron oxide taken up per cell by each cell lines, for an added concentration of 23.5 μg/ml and an incubation time of 24 h, presented as mean ± SEM. (n = 3)

The mass of iron oxide taken up per cell for all cell lines was calculated and shown in Fig. 2. Uptake was detected in all cell lines and showed cell line specific variability with HEPG2 cells having the highest level of SPION uptake, with a mass of 0.012 ± 0.005 pg per cell. In all other cell lines, SPION uptake was typically an order of magnitude smaller.

### SPION-induced DNA damage

**Fig. 3:**
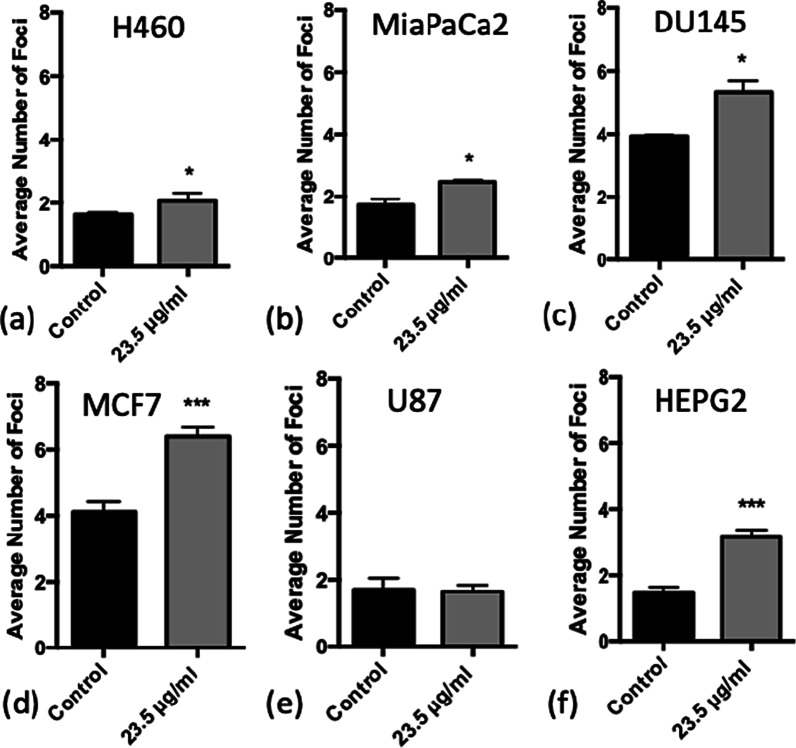
Average number of DNA damage foci counted for each cell line when treated with 23.5 μg/ml SPIONs in the absence of radiation, with 24 h incubation. **a** H460, **b** MiaPaCa2, **c** DU145, **d** MCF7, **e** U87 and **f** HEPG2. (n = 3) Presented as mean ± SD with statistical significance represented as; *P* < 0.05; *; *P* < 0.001: **; *P* < 0.0001: ***

To investigate the levels of DNA damage caused by SPION treatment alone, the levels of 53BP1 foci were measured in unirradiated control samples as shown in Fig. 3.

Significant increases in DNA damage were observed in all cell lines with the exception of U87 cells, suggesting U87 cells may be less sensitive to the biological effects of SPIONs. MCF7 and HEPG2 cells showed the most significant increase in DSBs, with an increase of 2.28 ± 0.32 and 1.70 ± 0.22 foci per cell for 23.5 μg/ml SPIONs respectively, which may be as a result of the uptake results in Fig. 1. However, DU145 and MCF7 cells had a larger number of foci, but this was due to the higher levels of background DNA damage measured in control samples. These results indicate a small inherent toxicity of SPIONs to cancer cell lines, which are worthwhile repeating for normal cell lines.

### Initial DNA damage in irradiated cells with SPIONs

**Fig. 4:**
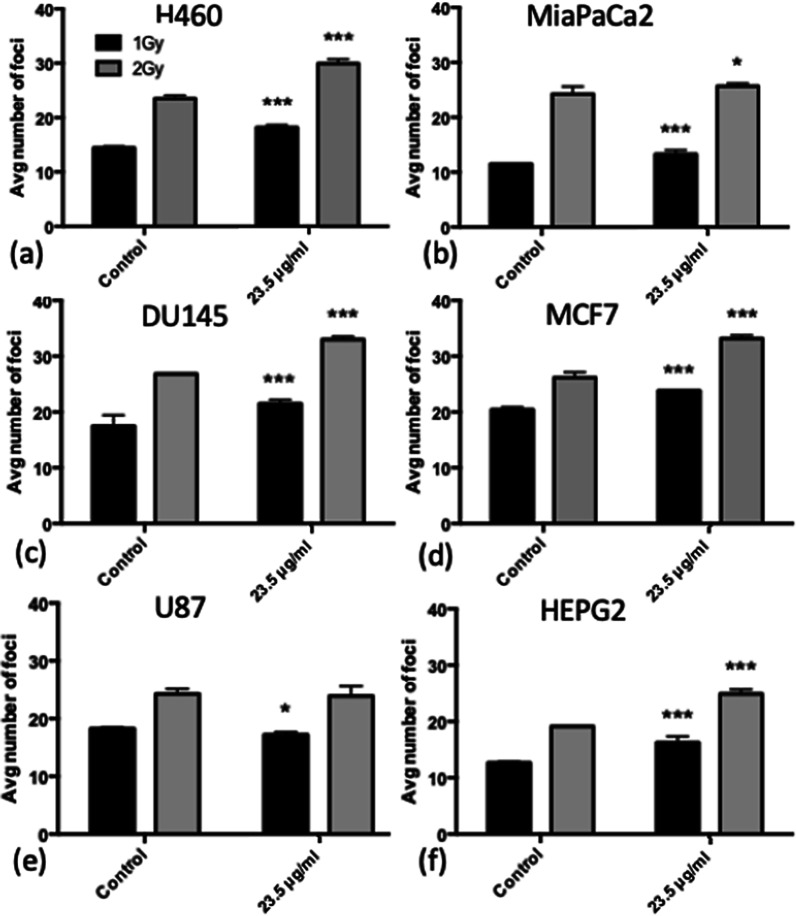
Average number of DNA damage foci counted for cells treated with 23.5 μg/ml SPIONs 1 h after exposure to 1 Gy or 2 Gy of X-rays. **a** H460, **b** MiaPaCa2, **c** DU145, **d** MCF7, **e** U87 and **f** HEPG2. (n = 3) Presented as mean ± SD with statistics represented as; *P* < 0.05: *; *P* < 0.001: **; *P* < 0.0001: ***

The number of DSB foci per cell was determined at doses of 1 and 2 Gy in the presence and absence of SPIONs at 1 h post-irradiation, as shown in Fig. 4. There is a significant increase in DNA damage at both 1 Gy and 2 Gy exposure, for all cell lines except for U87 cells when exposed to 23.5 μg/ml SPIONs compared with controls which were not treated with SPIONs, ranging from 5.1% for DU145 cells at 2 Gy to 30.1% increase for HEPG2 cells at 2 Gy, 1 h post-irradiation. This indicates that there is a radiosensitising effect of SPIONs in terms of initial DNA damage when combined with 225 kVp X-rays for the majority of cell lines. The ratios of DNA damage between cells treated with radiation and those not exposed to radiation, all treated with SPIONs (between Figs. 3 and 4) vary between 3.7–11.2 for 1 Gy and 5.2–17.2 for 2 Gy. The number of foci does not increase proportionally with the increased dose between 1 and 2 Gy doses, which may be due to overlapping foci being unable to be resolved as single foci, or cells detaching at higher doses, altering the interpretation of the data. However, this would not have a large effect as survival curves show only a small fraction of cells are killed at a dose of 2 Gy.

### Evaluation of DSB repair

**Fig. 5:**
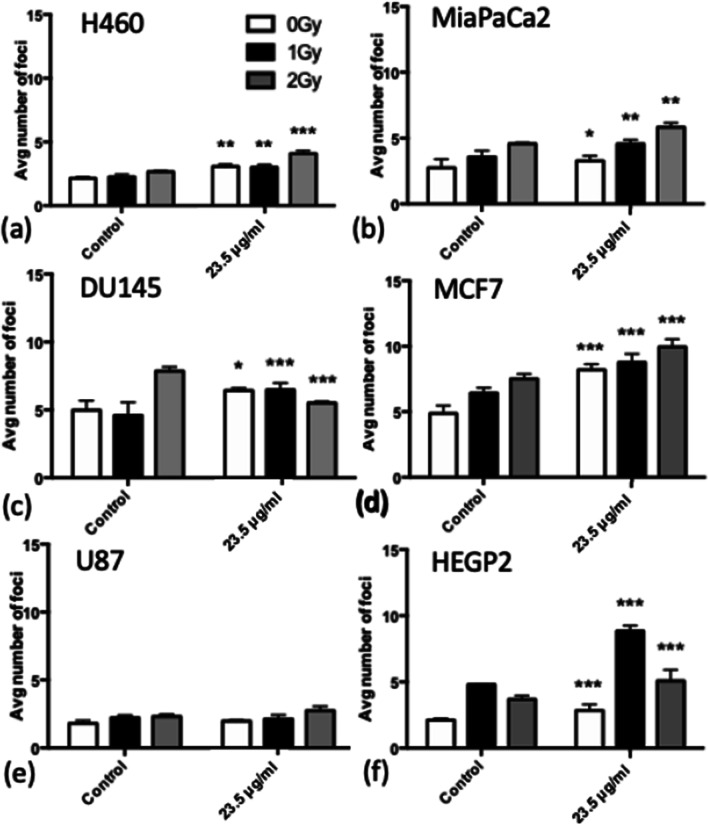
Average number of DNA damage foci counted for each cell line when treated with 23.5 μg/ml SPIONs and doses of 0–2 Gy measured at 24 h after irradiation. **a** H460, **b** MiaPaCa2, **c** DU145, **d** MCF7, **e** U87 and **f** HEPG2. (n = 3) Presented as mean ± SD with statistics represented as; *P* < 0.05: *; *P* < 0.001: **; *P* < 0.0001: ***

To determine whether the increase in DNA damage caused by the combination of SPIONs with radiation is repairable, the number of foci per cell was counted for the 24 h time point after exposure to doses of 0–2 Gy (Fig. 5). All of the foci yields are lower than in Fig. 3, and so, for all cell lines, the DNA damage is repairable to some extent, with the percentage repair ranging from 45.6–90.5% across all cell lines for doses of 1 Gy and 2 Gy, not accounting for background damage. However, there is a significant increase in DNA damage remaining at 24 h for all cell lines for all doses after SPION treatment, except for U87 cells and also DU145 cells at 2 Gy, when compared with controls. The ratios of DNA damage between cells treated with radiation and those not exposed to radiation, all treated with SPIONs vary between 0.97–3.11 for 1 Gy and 0.85–1.79 for 2 Gy. HEPG2 cells saw a much larger number of residual foci at 1 Gy than at 2 Gy, which requires further investigation.

### DNA damage vs uptake

**Fig. 6:**
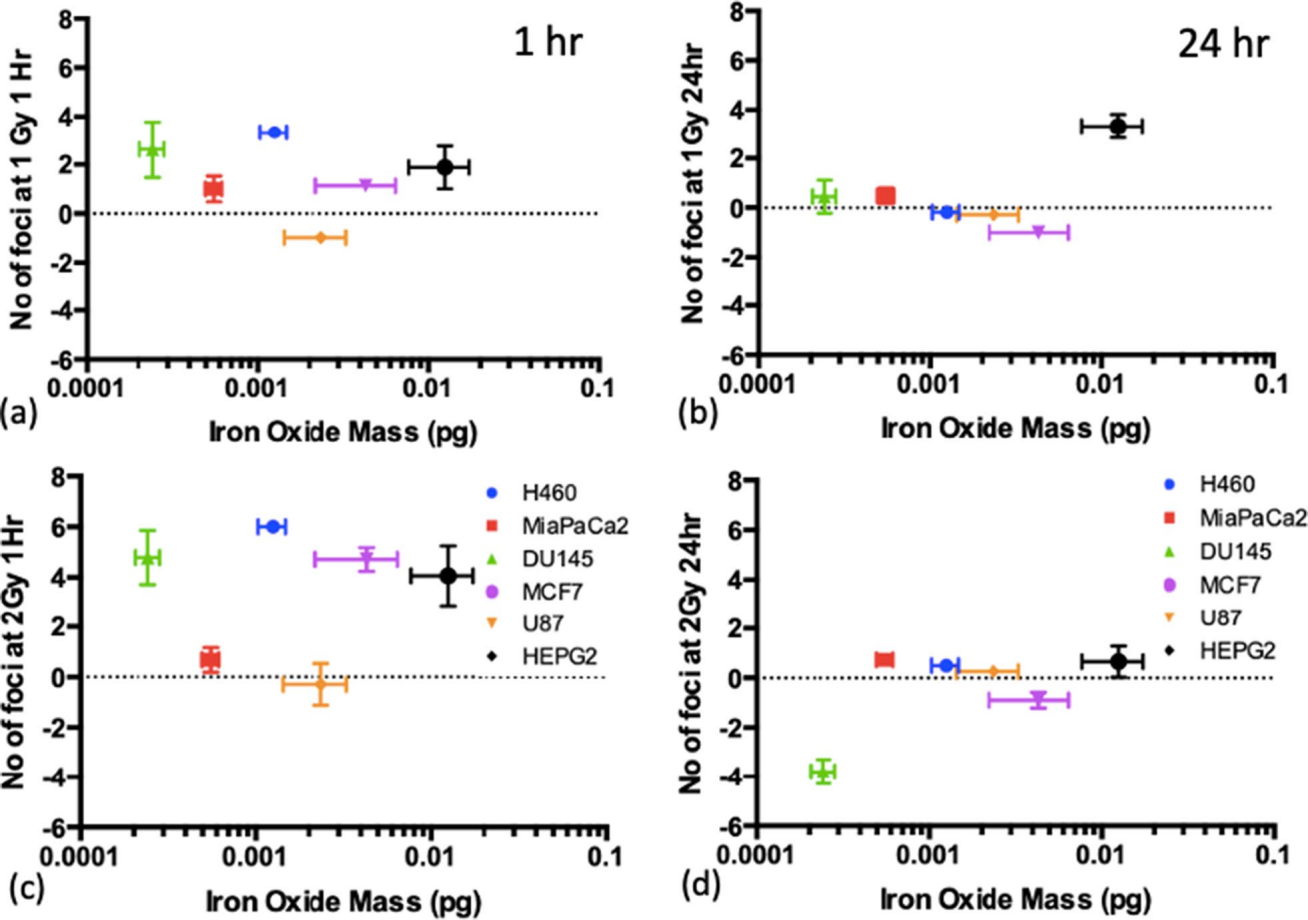
Mass of iron oxide detected per cell for each cell line compared to the number of foci counted, corrected for controls, and also corrected for the number of foci for cells without SPIONs, at the 1 h timepoint (left) and 24 h timepoint (right) after combined exposure with SPIONs and 225 kVp X-rays for; **a**, **c** 1 Gy and **b**, **d** 2 Gy. Presented as mean ± SD. Spearman Correlation coefficient, ρ, is indicated on each graph

Figure 6 show the levels of DNA damage measured at the 1 h and 24 h timepoints for doses of 1 Gy and 2 Gy compared with the ICP-MS uptake measurements reported in Fig. 2, to determine whether there is a correlation between the amount of SPIONs being taken up by the cells and the associated radiosensitivity, using the Spearman coefficient. The foci numbers had background levels of DNA damage subtracted (the levels of foci at 0 Gy) to present the radiosensitisation only, and not inherent toxicity, as well as subtracting the number of foci counted when no SPIONs were present.

For the 1 h timepoint, in particular, DU145 cells had one of the highest levels of foci at each dose point, and yet they also had the lowest level of uptake, with the smallest mass of iron oxide detected. No strong correlation (greater than + / − 0.7) was found between the mass of iron oxide and the radiosensitivity of the cell lines at either timepoint, suggesting that radiosensitisation is mostly due to cell line dependant toxicity, rather than the levels of uptake.

### Cell survival and sensitisation enhancement ratios

**Fig. 7:**
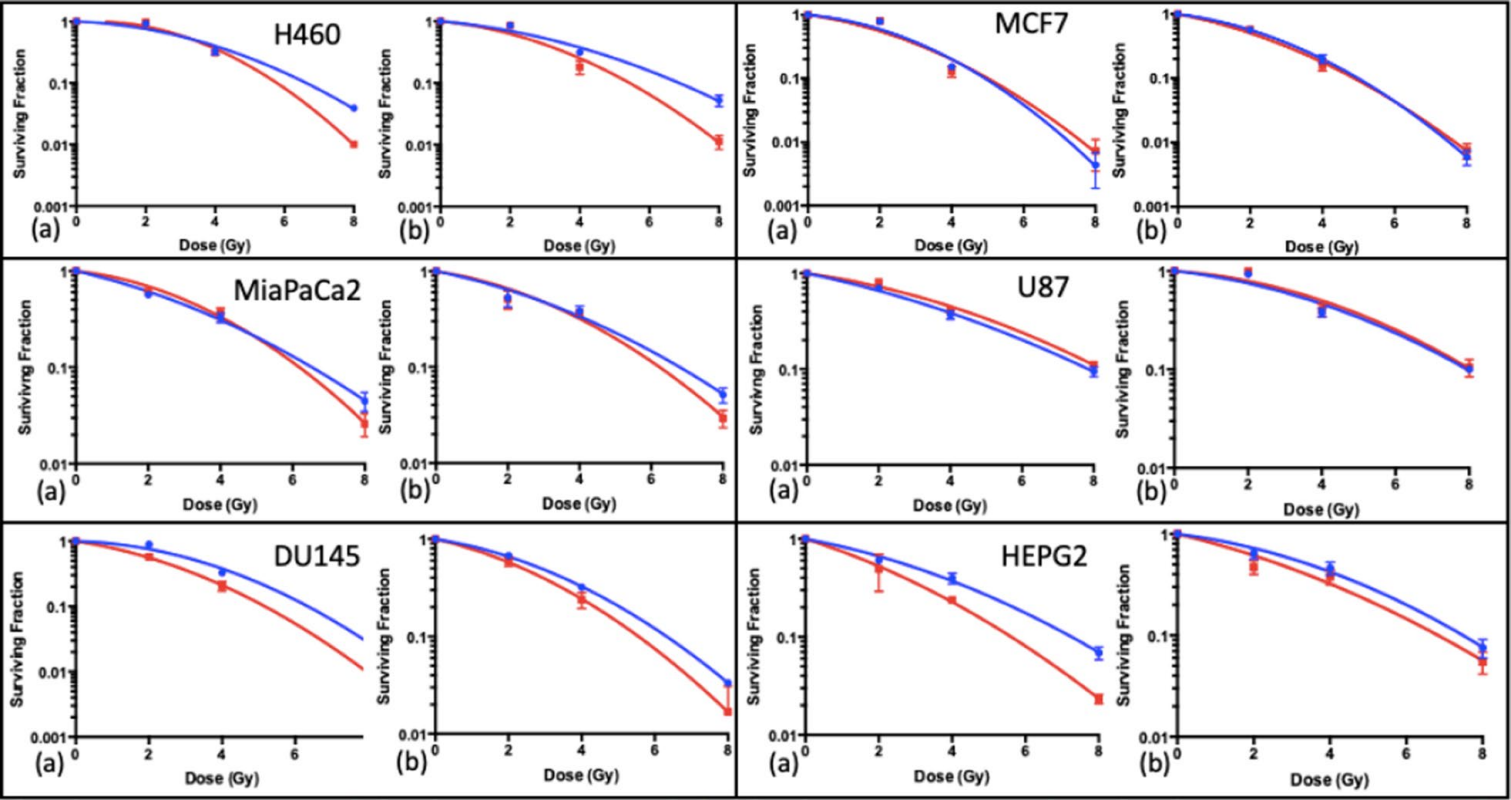
Clonogenic cell survival curves fitted with the linear quadratic model for the combination of 225 kVp X-rays with 23.5 μg/ml SPIONs, for a 1 h exposure time; **a** 1 h and **b** 24 h exposure times. (n = 3) Presented as mean ± SD

The clonogenic assay was performed for SPION exposures of both 1 h and 24 h prior to irradiation. The purpose was to investigate the impact of SPIONs on clonogenic cell survival, and the impact of exposure time. Graphs of both 1 h and 24 h exposure times are shown in Fig. 7 respectively, with corresponding $\alpha /\beta$ ratios and SERs shown in Tables 1 and 2 respectively.

**Table 1:** $\alpha /\beta$ ratios and SERs SERs taken from the corresponding linear quadratic curves in Fig. 6 for a 1 h exposure time. The SPION graph for H460 cells was unable to generate an accurate *α* value, which was constrained to be > 0

Cell line	SPION concentration							
	Control			23.5 μg/ml				
	$\alpha$(Gy^−1^)	$\beta$(Gy^−2^)	$\alpha /\beta$(Gy)	$\alpha$(Gy^−1^)	$\beta$(Gy^−2^)	$\alpha /\beta$(Gy)	SER for 8 Gy	SER for 10% SF
H460	0.049 ± 0.047	0.045 ± 0.006	1.08 ± 0.61	0	0.081 ± 0.005	0	1.18 ± 0.10	1.45 ± 0.36
MiaPaCa2	0.198 ± 0.049	0.024 ± 0.007	8.33 ± 1.81	0.094 ± 0.059	0.045 ± 0.092	2.09 ± 0.79	1.09 ± 0.10	1.05 ± 0.47
DU145	0.038 ± 0.064	0.051 ± 0.009	0.74 ± 0.72	0.196 ± 0.013	0.048 ± 0.002	4.06 ± 0.17	1.17 ± 0.07	1.23 ± 0.19
MCF7	0.118 ± 0.122	0.071 ± 0.017	1.67 ± 0.95	0.186 ± 0.132	0.055 ± 0.018	2.77 ± 1.03	0.95 ± 0.39	0.98 ± 0.30
U87	0.180 ± 0.031	0.015 ± 0.004	12.35 ± 2.45	0.122 ± 0.030	0.019 ± 0.004	6.23 ± 1.18	0.96 ± 0.21	0.94 ± 0.17
HEPG2	0.161 ± 0.042	0.021 ± 0.006	7.51 ± 1.61	0.272 ± 0.076	0.025 ± 0.011	11.08 ± 3.26	1.28 ± 0.03	1.31 ± 0.32

**Table 2:** $\alpha /\beta$ ratios and SERs taken from the corresponding linear quadratic curves in Fig. 7, for a 24 h exposure time

Cell line	SPION concentration							
	Control			23.5 μg/ml				
	$\alpha$(Gy^−1^)	$\beta$(Gy^−2^)	$\alpha /\beta$(Gy)	$\alpha$(Gy^−1^)	$\beta$(Gy^−2^)	$\alpha /\beta$(Gy)	SER for 8 Gy	SER for 10% SF
H460	0.117 ± 0.057	0.032 ± 0.008	3.68 ± 1.16	0.125 ± 0.084	0.055 ± 0.012	2.26 ± 0.92	1.27 ± 0.03	1.25 ± 0.30
MiaPaCa2	0.174 ± 0.059	0.024 ± 0.008	7.11 ± 1.96	0.133 ± 0.062	0.038 ± 0.009	3.47 ± 1.04	1.11 ± 0.10	1.09 ± 0.26
DU145	0.136 ± 0.009	0.036 ± 0.001	3.75 ± 0.15	0.196 ± 0.009	0.039 ± 0.001	4.98 ± 0.17	1.12 ± 0.01	1.14 ± 0.04
MCF7	0.165 ± 0.057	0.060 ± 0.008	2.77 ± 0.82	0.251 ± 0.065	0.045 ± 0.009	5.52 ± 1.12	0.97 ± 0.20	1.02 ± 0.18
U87	0.095 ± 0.047	0.025 ± 0.007	3.84 ± 1.25	0.061 ± 0.064	0.028 ± 0.009	2.16 ± 1.37	0.99 ± 0.35	0.98 ± 0.26
HEPG2	0.108 ± 0.041	0.027 ± 0.006	4.06 ± 1.02	0.206 ± 0.085	0.019 ± 0.012	10.70 ± 4.57	1.08 ± 0.31	1.09 ± 0.31

Statistical analysis was carried out for this data using the Extra Sum-of-Squares F-Test. It was found that all cell lines, except for MCF7 and U87 cells, had a significant change in the linear quadratic curve for the 1 h exposure, and for the 24 h exposure, a significant change was seen for H460 and DU145 cells (*P* < 0.05). This indicates a cell specific radiosensitisation that is dependent on the exposure time.

The SER calculated using the clonogenic results for all cell lines for both 1 h and 24 h exposure is shown in Tables 1 and 2, ranging from 0.95 ± 0.39 for MCF7 cells to 1.28 ± 0.03 for HEPG2 cells. This suggests radiosensitising effects from SPIONs for all cell lines apart from MCF7 and U87 cells. However, the predicted DEF calculated by taking the ratio of absorption coefficients of medium compared with medium plus iron oxide was found to be just 1.000138, suggesting little to no physical radiosensitisation was expected for the clonogenic assay, as explained in detail in the methods. It is worth noting that survival curves are non-linear and so not directly comparable to the DEF, but allow for an indication of the magnitude of physical and biological enhancement as a contribution to radiosensitisation.

**Fig. 8:**
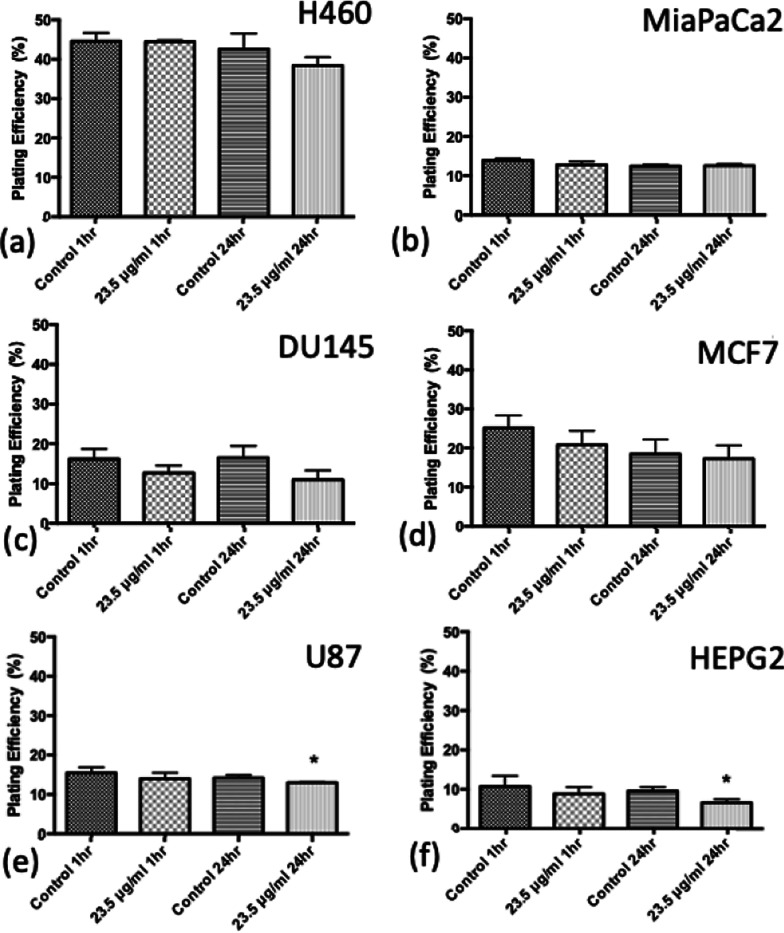
Plating efficiency for cell lines in the absence of radiation, for control samples and 23.5 μg/ml SPIONs for both 1 h and 24 h exposure times. **a** H460, **b** MiaPaCa2, **c** DU145, **d** MCF7, **e** U87 and **f** HEPG2. (n = 3) Presented as mean ± SD with statistics represented as; *P* < 0.05: *; *P* < 0.001: **; *P* < 0.0001: ***

To investigate further the toxic effect of SPIONs, the plating efficiencies at for the 1 h and 24 h exposure to SPIONs have been plotted for 0 Gy values without irradiation in Fig. 8. The only significant decrease in plating efficiency occurred for the 24 h exposure for U87 and HEPG2 cells (*P* < 0.05). This suggests that the decrease in cell survival seen is due to radiosensitisation and not the inherent toxicity of the SPIONs.

### In vivo model

**Fig. 9:**
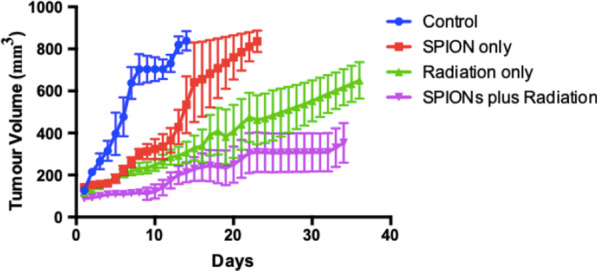
Tumour Volume with time for in vivo experiment for 4 subgroups; control, SPION only, radiation only, and SPIONs plus radiation, taken as the median value across all mice, using linear interpolation for days between measurements. Presented as mean ± SEM

**Fig. 10:**
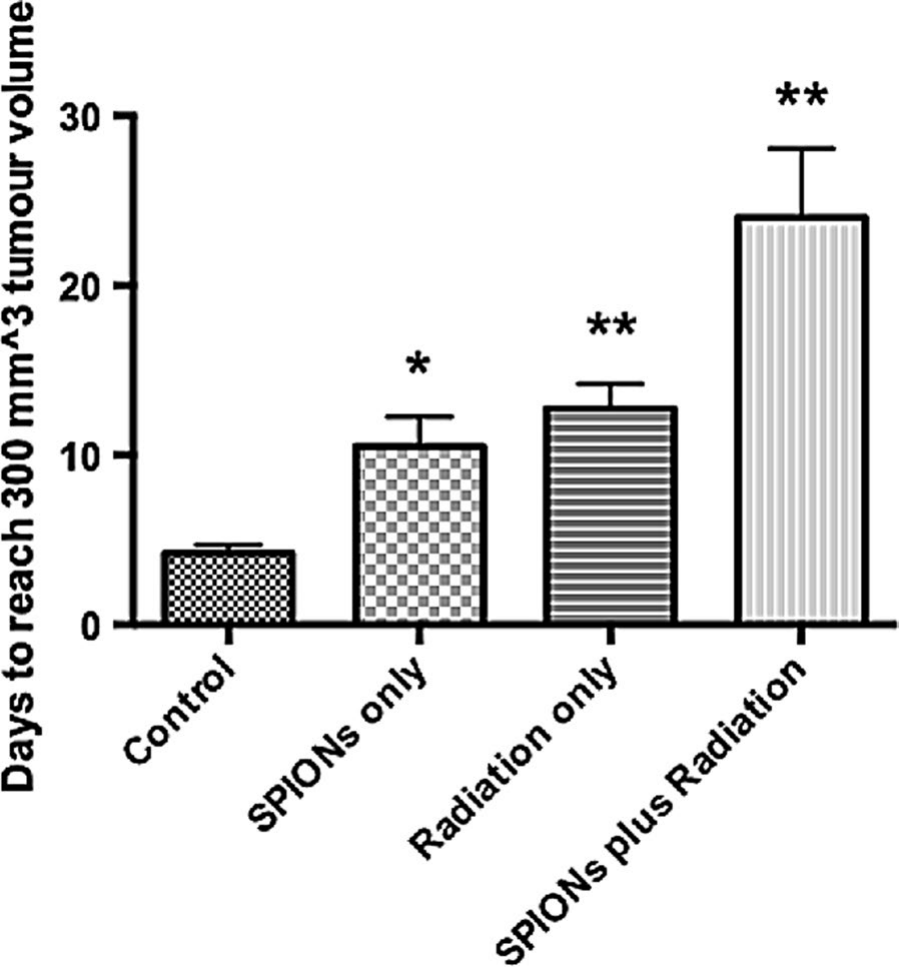
Average time (days) for tumour volume to reach 300 mm^3^ for the 4 subgroups; control, SPIONs only, radiation only, and SPIONs plus radiation. Presented as mean ± SEM with statistics represented as; *P* < 0.05: *; *P* < 0.001: **; *P* < 0.0001: ***

The observed changes in tumour growth for the different experimental groups are shown in Fig. 9. The median tumour volume was taken using linear interpolation for days between measurements. To quantify tumour growth delay, the time taken for each subgroup to reach a tumour size of 300 mm^3^ is represented in Fig. 10, with statistics carried out using the Student’s *t* Test. This represents a significant decrease in tumour growth for all subgroups when compared with controls. SPIONs alone delayed tumour growth, with the combination of SPIONs and radiation having the largest impact, with an average time to reach a 300 mm^3^ tumour volume of 24 ± 4 days, compared with 4.2 ± 0.5 days for controls. The average times for the SPION only and the radiation only group were 10.5 ± 1.8 days and 12.8 ± 1.5 days respectively.

## Discussion

This study aimed to assess the radiosensitising properties of SPIONs as potential theranostic agents. An inherent increased DNA damage and decreased survival was observed with 23.5 μg/ml SPIONs in all cell lines excluding U87 cells, for a 24 h incubation time. The most significant effects were seen in MCF7 and HEPG2 cells (*P* < 0.0001). These data are in contrast to a previous study by Khoshgard et al. which reported no cytotoxicity for SPIONs following 24 h exposures in MCF7 and HeLa cells, using an MTT assay [67]. Also, the inherent cytotoxicity observed in our study did not correlate DNA damage, suggesting that it is possible that in cell lines other than U87 and HEPG2, any decrease in cell survival post-irradiation was due to radiosensitisation, along with other possible causes of DNA damage.

The impact of combinations of SPIONs with radiation on DNA damage yields is presented in Fig. 4. All cell lines saw a significant increase in damage at 1 Gy, and all cell lines except U87 cells at 2 Gy, 1 h post-irradiation. It suggests an increase in DNA damage driven by SPIONs alone on the cells, as well as a radiosensitising effect. At the 24 h timepoint, once again, all cell lines except for U87 cells see a significant increase in DNA damage at 1 Gy. However, significant increases were only seen for H460, MiaPaCa2, MCF7 and HEPG2 cells at 2 Gy. DNA damage decreased at 2 Gy for DU145 cells. While still significant, HEPG2 cells saw higher levels of DNA damage at 1 Gy than 2 Gy, however the foci numbers at the 24 h timepoint are small, and so the differences in the residual levels of DNA damage are small, suggesting repair is effective.

No strong correlation was found between ICP-MS uptake and DNA damage at the both the 1 h and 24 h timepoint, which contrasts previous work with a gadolinium contrast agent (Dotarem) in which there was a positive correlation [68]. This difference may be due to the higher concentrations of Dotarem used in comparison to the SPIONs, and also that, for iron and irradiation with 100 keV beams, photoelectrons can have energy of around 90 keV, providing a range in water of > 20 microns, which is greater than the dimensions of a cell. Cell lines had taken up varying quantities of SPIONs (measured by ICP-MS), but the cell-specific sensitivity of the cell to the SPIONs seems not be solely due to the concentration within the cell, as demonstrated by U87 cells. Ahmad et al. appears to disagree with these results, finding a positive correlation between levels of DNA damage and uptake with ICP-MS, for Iron Oxide nanoparticles with a mean diameter of 140 ± 4 nm at a concentration of 0.5 mg/ml, which is much higher than the concentration used for this study [69]. However, Ahmad et al. finds a complex relationship between uptake and radiosensitivity, as there is a negative relationship between dose enhancement and uptake, and this was carried out for 6 MV, not the 225 kVp used in this study. Increasing the SPION concentration may impact the relationship between DNA damage through both physical and biological dose enhancement, therefore further work is necessary to determine this relationship.

Exposure times for the DNA damage and clonogenic assay were 24 h exposure (with the clonogenic assay also having a 1 h exposure time). All cell lines except for MCF7 and U87 cells showed some level dose enhancement by the SER, correlating directly for U87 with the DNA damage results for the 1 h timepoint, yet appears to disagree with the DNA damage seen for MCF7 cells. However, a significant change in the survival curve for 24 h exposure was seen only for H460 and DU145 cells, suggesting higher levels of DNA damage may be repairable in certain cell lines.

The predicted DEF was found only to be 1.0001, compared with SERs found to vary up to 1.28 for HEPG2 cells for 1 h exposure time to SPIONs. This emphasises an important point, that the radiosensitisation of nanoparticles have 2 factors; the physical dose enhancement due to an increase in secondary electrons, and the biological radiosensitisation. For SPIONs, the biological effects are much more prominent, and has implications on the biological functions, likely increasing oxidative stress by increasing levels of ROS and hydroxyl radicals [12, 15, 16], depending on variables such as the concentration and the energy of the radiation.

Kirakli et al. investigated the impact of SPIONs on radiosensitisation in vitro, through both clonogenic assays and metabolic activity assays for 3 cell lines; MCF7, MDA-MB-231 and MDAH-2774 [56]. The SER, which they termed nanoparticle-mediated enhancement ratio (NER) was greatest at 2 Gy, and the radiosensitising effects had diminished by 8 Gy [56]. However, Kirakli et al. performed the study using 6 MV X-rays and there may be cell-type dependent effects. Overall, there is a paucity of data in regard to the radiosensitisation of SPIONs in-vitro, in particular for the kV energy range and specific cell types.

For the in vivo experimentation, all subgroups showed a significant decrease in the rate of tumour growth (*P* < 0.05), measured as the time for the tumour volume to reach 300 mm^3^. This shows that SPIONs alone impaired the ability for the tumour cells to grow and divide (Fig. 9), however this disagrees with Fig. 8, in which no decrease in clonogenic viability was found. In combination with radiation, SPIONs have a greater impact on tumour growth (Fig. 10), however, it must be noted that the small sample size can lead to uncertain statistics, and should therefore be used only as a guide. SPIONs may be leading to the production of higher levels of ROS and greater DNA damage, as suggested by Dayem et al. [11] Iron oxide has the potential to cause an increase in ROS production through the Haber–Weiss reaction, in which iron ions generate hydroxyl free radicals [55, 70]. The results can be compared with a similar in vivo experiment with AGuIX, a gadolinium sub 5 nm nanoparticle currently in a Phase 1 clinical trial [46]. Dufort et al. performed the in vivo experiment on rats bearing gliosarcoma tumours, demonstrating an increase in the survival time of the rats with microbeam radiation, up to 6.4 keV [71]. The median survival time was found to increase by a factor of 3.10 and 4.75 for AGuIX injections 1 h and 24 h prior to irradiation respectively, compared with controls [71], compared with 5.71 for SPIONs plus radiation in this study. The in vivo results validate the in vitro methods, in which H460 cells consistently indicated toxicity as well as a decrease in cell survival and an increase in DNA damage.

One limitation of comparing the in vitro and in vivo experimentation of this study is in the difference between concentrations of SPIONs used and the different incubation times. Due to the nature of the in vivo experiment being a pilot study, it was unknown for how long the SPIONs would remain inside the tumour, therefore a shorter incubation time was chosen. Also, the number of cells in a solid tumour is much greater than that used for the in vitro experiments, hence the much higher concentration of SPIONs used. The fact that the SPIONs were injected intratumourally means also that the EPR effect was not tested in this experiment. These differences should be considered when directly comparing these results.

It is also important to note the difference between the kVp source used for this investigation and the MV energies which are conventionally used for radiotherapy. kVp energies are dominated by the photoelectric effect which is proportional to Z^3^ compared with the Compton effect for MV energies which is proportional to Z [59], suggesting that radiosensitisation from the nanoparticles would be greater for kVp energies. However, the largest differences in the ratios of absorption coefficients occur in the region below 100 keV [60] which are strongly attenuated by the 2 mm Cu filter used. In particular, the k-edge of iron oxide is < 10 keV, further reducing the effect [60]. Whilst it is important to acknowledge the differences in the physical dose enhancement caused by the differences in energy, some comparison can be made due to the biological effects being of greater impact.

DLS measurements indicated a hydrodynamic radius of 124 ± 6 nm for the SPIONs, when averaged across a range of concentrations. This is much greater than the 5 nm nominal diameter reported by the manufacturer and may influence the mechanism by which the SPIONs are taken up by cells, although the exact mechanism of uptake is not known. This result is surprising, although comparable with previously published work where Lanier et al. reported differences of the same order of magnitude for hydrodynamic radius’ compared with physical nanoparticle size as quoted by a manufacturer [72]. This large hydrodynamic radius may suggest the possibility of aggregation of the nanoparticles, however, the measurements also indicated that the SPIONs are predominantly mono-disperse and do not aggregate. Measurements for polydispersity (see Additional file 1) which indicate the distribution of nanoparticle sizes, show a perfect sigmoidal curve starting at ~ 0.9 with a steep decay suggesting no signs of aggregation. Aggregation has been discussed in relation to magnetic nanoparticles by Gutiérrez et al., with evidence that aggregation impacts the interaction of the nanoparticles with cells [73]. What is not clear from this study, however, is whether aggregation would occur when the SPIONs are exposed to biological fluids, possibly resulting in protein coronas surrounding the nanoparticles (74).

From this initial study, the next step is to investigate radiosensitising effects with the superparamagnetic properties of SPIONs by combining radiation exposures with an alternating magnetic field to mimic scenarios relevant to MR-Linac exposures, which are expanding in use clinically. Also, further studies using non-cancer cell lines and MV energies should be carried out, and an investigation of the impact of varying SPION concentration on radiosensitisation. To determine in greater detail how radiosensitisation occurs, the mechanism of uptake should also be investigated.

## Conclusion

This study defines the radiosensitising properties of SPIONs in combination with kVp X-rays for 6 different cancer cell lines in vitro, as well as for the first time, in vivo with a H460 lung xenograft model. This has been validated using both clonogenic assays and DNA damage immunofluorescence assays, with further investigations into the uptake of SPIONs into the cells, using ICP-MS, alongside DLS to determine the hydrodynamic radius of the SPIONs. This study finds strong evidence that SPIONs cause radiosensitisation, with a significant increase in DSBs, but also a significant change in the clonogenic cell survival. However, no strong correlation was found between the mass of iron oxide taken up into the cells and the levels of radiosensitisation, 1 h post-irradiation, suggesting physical dose enhancement is not the primary mechanism of sensitisation, although further studies are needed to verify this.

## Supplementary Information

**Additional file 1.** Graph of time against correlation coefficient for 3 repeat measurements. It is a near perfect sigmoidal curve starting at 0.9 on the y-axis suggesting no aggregation of nanoparticles.

**Additional file 2.** Graph of the filtered x-ray spectrum used for the in vitro experimentation.

**Additional file 3.** Graph of energy against DEF for the range of energies in the x-ray spectrum used for in vitro experimentation.

## Data Availability

The datasets during and/or analysed during the current study available from the corresponding author on reasonable request.

## References

[1] Bekelman JE, Lee WR (2017). Six questions to ask before we shorten radiation treatments for intact prostate cancer. Int J Radiat Oncol Biol Phys.

[2] Lee WR (2016). Hypofractionation for prostate cancer: tested and proven. Lancet Oncol.

[3] Symonds P, Jones GDD (2019). FLASH radiotherapy: The next technological advance in radiation therapy?. Clin Oncol.

[4] Bourhis J, Montay-Gruel P, Gonçalves Jorge P, Bailat C, Petit B, Ollivier J (2019). Clinical translation of FLASH radiotherapy: Why and how?. Radiother Oncol.

[5] Vozenin MC, Hendry JH, Limoli CL (2019). Biological benefits of ultra-high dose rate FLASH radiotherapy: sleeping beauty awoken. Clin Oncol.

[6] Sharma RA, Plummer R, Stock JK, Greenhalgh TA, Ataman O, Kelly S (2016). Clinical development of new drug-radiotherapy combinations. Nat Rev Clin Oncol.

[7] Schuemann J, Bagley AF, Berbeco R, Bromma K, Butterworth KT, Byrne H, et al. Roadmap for metal nanoparticles in radiation therapy: current status translational challenges, and future directions. Phys Med Biol. 2020;(May). 10.1088/1361-6560/ab9159 32380492

[8] Rabus H, Gargioni E, Li WB, Nettelbeck H, Villagrasa C. Determining dose enhancement factors of high-Z nanoparticles from simulations where lateral secondary particle disequilibrium exists. Phys Med Biol. 2019;64(15). 10.1088/1361-6560/ab31d4 31300616

[9] Roeske JC, Nuñez L, Hoggarth M, Labay E, Weichselbaum RR (2007). Characterization of the theorectical radiation dose enhancement from nanoparticles. Technol Cancer Res Treat.

[10] Butterworth KT, McMahon SJ, Currell FJ, Prise KM (2012). Physical basis and biological mechanisms of gold nanoparticle radiosensitization. Nanoscale.

[11] Dayem AA, Hossain MK, Lee S Bin, Kim K, Saha SK, Yang GM, et al. The role of reactive oxygen species (ROS) in the biological activities of metallic nanoparticles. Int J Mol Sci 2017;18(1):1–21. 10.3390/ijms18010120 PMC5297754 28075405

[12] Rosa S, Connolly C, Schettino G, Butterworth KT, Prise KM. Biological mechanisms of gold nanoparticle radiosensitization. Cancer Nanotechnol. 2017;8(1). 10.1186/s12645-017-0026-0 PMC5288470 28217176

[13] Abdul Rashid R, Zainal Abidin S, Khairil Anuar MA, Tominaga T, Akasaka H, Sasaki R (2019). Radiosensitization effects and ROS generation by high Z metallic nanoparticles on human colon carcinoma cell (HCT116) irradiated under 150 MeV proton beam. OpenNano..

[14] Shrivastava R, Kushwaha P, Bhutia Y, Flora S (2016). Oxidative stress following exposure to silver and gold nanoparticles in mice. J Toxicol Ind Heal.

[15] Manke A, Wang L, Rojanasakul Y. Mechanisms of nanoparticle-induced oxidative stress and toxicity. Biomed Res Int. 2013;2013. 10.1155/2013/942916 PMC3762079 24027766

[16] Mateo D, Morales P, Avalos A, Haza AI. Oxidative stress contributes to gold nanoparticle-induced cytotoxicity in human tumor cells. Toxicol Mech Methods. 2014;24. 10.3109/15376516.2013.869783 24274460

[17] Singh N, Jenkins GJS, Asadi R, Doak SH (2010). Potential toxicity of superparamagnetic iron oxide nanoparticles (SPION). Nano Rev.

[18] Park J, Choi Y, Chang H, Um W, Ryu JH, Kwon IC (2019). Alliance with EPR effect: combined strategies to improve the EPR effect in the tumor microenvironment. Theranostics.

[19] Fang J, Nakamura H, Maeda H (2011). The EPR effect: Unique features of tumour blood vessels for drug delivery, factors involved, and limitations and augmentation of the effect. Adv Drug Deliv Rev.

[20] Torchilin V (2011). Tumor delivery of macromolecular drugs based on the EPR effect. Adv Drug Deliv Rev.

[21] Grobmyer SR, Moudgil BM, Walker JM. Cancer nanotechnology methods and protocols. Springer; 2010. 10.1007/978-1-60761-609-2.

[22] Hanžić N, Horvat A, Bibic J, Unfried K, Jurkin T, Drazic G (2018). Syntheses of gold nanoparticles and their impact on the cell cycle in breast cancer cells subjected to megavoltage X-ray irradiation. Mater Sci Eng.

[23] Lechtman E, Mashouf S, Chattopadhyay N, Keller BM, Lai P, Cai Z (2013). A Monte Carlo-based model of gold nanoparticle radiosensitization accounting for increased radiobiological effectiveness. Phys Med Biol.

[24] Hainfeld JF, Smilowitz HM, O’connor MJ, Dilmanian FA, Slatkin DN (2013). Gold nanoparticle imaging and radiotherapy of brain tumors in mice. Nanomedicine.

[25] Miladi I, Alric C, Dufort S, Mowat P, Dutour A, Mandon C (2014). The in vivo radiosensitizing effect of gold nanoparticles based MRI contrast agents. Small.

[26] Zhang X, Wang H, Coulter JA, Yang R (2018). Octaarginine-modified gold nanoparticles enhance the radiosensitivity of human colorectal cancer cell line LS180 to megavoltage radiation. Int J Nanomed.

[27] Chattopadhyay N, Cai Z, Kwon YL, Lechtman E, Pignol JP, Reilly RM (2013). Molecularly targeted gold nanoparticles enhance the radiation response of breast cancer cells and tumour xengrafts to X-radiation. Breast Cancer Res Treat.

[28] Roa W, Zhang X, Guo L, Shaw A, Hu X, Xiong Y, et al. Gold nanoparticle sensitize radiotherapy of prostate cancer cells by regulation of the cell cycle. Nanotechnology. 2009;20(37). 10.1088/0957-4484/20/37/375101 19706948

[29] Zhang X, Xing J, Cehn J, Ko L, Amanie J, Gulavita S (2008). Enhanced radiation sensitivity in prostate cancer by gold-nanoparticles. Clin Investig Med.

[30] Chang MY, Shiau AL, Chen YH, Chang CJ, Chen HHW, Wu CL (2008). Increased apoptotic potential and dose-enhancing effect of gold nanoparticles in combination with single-dose clinical electron beams on tumor-bearing mice. Cancer Sci.

[31] Butterworth KT, Coulter JA, Jain S, Forker J, McMahon SJ, Schettino G, et al. Evaluation of cytotoxicity and radiation enhancement using 1.9nm gold particles: potential application for cancer therapy. Nanotechnology. 2010;21(29). 10.1088/0957-4484/21/29/295101 PMC3016629 20601762

[32] Jain S, Coulter J, Butterworth K, Hounsell A, McMahon S, Hyland W (2014). Gold nanoparticle cellular ultake, toxicity and radiosensitisation in hypoxic conditions. Radiother Oncol.

[33] Joh DY, Sun L, Stangl M, Al Zaki A, Murty S, Santoiemma PP, et al. Selective targeting of brain tumors with gold nanoparticle-induced radiosensitization. PLoS ONE. 2013;8(4). 10.1371/journal.pone.0062425 PMC3640092 23638079

[34] Chanda N, Kan P, Watkinson LD, Shukla R, Zambre A, Carmack TL (2010). Radioactive gold nanoparticles in cancer therapy: therapeutic efficacy studies of GA-198AuNP nanoconstruct in prostate tumor-bearing mice. Nanomed Nanotechnol Biol Med.

[35] Miladi I, Duc L, Kryza D, Mowat P, Tillement O, Billotey C (2012). Biodistribution of ultra small gadolinium-based nanoparticles as theranostic agent. Appl Brain Tumors.

[36] Taupin F, Flaender M, Delorme R, Brochard T, Mayol J-F, Arnaud J (2015). Gadolinium nanoparticles and contrast agent as radiation sensitizers. Phys Med Biol.

[37] Delorme R, Taupin F, Flaender M, Ravanat JL, Champion C, Agelou M (2017). Comparison of gadolinium nanoparticles and molecular contrast agents for radiation therapy-enhancement. Med Phys.

[38] Kotb S, Detappe A, Lux F, Appaix F, Barbier EL, Plissonneau M (2016). Gadolinium-based nanoparticles and radiation therapy for multiple brain melanoma metastases: proof of concept before phase I trial. Theranostics.

[39] Le Duc G, Roux S, Paruta-Tuarez A, Dufort S, Brauer E, Marais A (2014). Advantages of gadolinium based ultrasmall nanoparticles vs molecular gadolinium chelates for radiotherapy guided by MRI for glioma treatment. Cancer Nanotechnol.

[40] Lux F, Sancey L, Bianchi A, Crémillieux Y, Roux S, Tillement O (2015). Gadolinium-based nanoparticles for theranostic MRI-radiosensitization. Nanomedicine.

[41] Li F, Li Z, Jin X, Liu Y, Zhang P, Li P (2019). Ultra-small gadolinium oxide nanocrystal sensitization of non-small-cell lung cancer cells toward X-ray irradiation by promoting cytostatic autophagy. Int J Nanomed.

[42] Detappe A, Kunjachan S, Rottmann J, Robar J, Tsiamas P, Korideck H (2015). AGuIX nanoparticles as a promising platform for image-guided radiation therapy. Cancer Nanotechnol.

[43] Štefančíková L, Lacombe S, Salado D, Porcel E, Pagáčová E, Tillement O (2016). Effect of gadolinium - based nanoparticles on nuclear DNA damage and repair in glioblastoma tumor cells. J Nanobiotechnol.

[44] Zhang DG, Feygelman V, Moros EG, Latifi K, Zhang GG (2014). Monte Carlo study of radiation dose enhancement by gadolinium in megavoltage and high dose rate radiotherapy. PLoS ONE.

[45] Luchette M, Korideck H, Makrigiorgos M, Tillement O, Berbeco R (2014). Radiation dose enhancement of gadolinium-based AGuIX nanoparticles on HeLa cells. Nanomed Nanotechnol Biol Med.

[46] Radiotherapy of Multiple Brain Metastases Using AGuIX (NANORAD2) [Internet]. ClinicalTrials.gov, U.S National Library of Medicine. [cited 2020 Apr 16]. https://clinicaltrials.gov/ct2/show/NCT03818386

[47] McMahon SJ, Paganetti H, Prise KM (2016). Optimising element choice for nanoparticle radiosensitisers. Nanoscale.

[48] Dulińska-Litewka J, Łazarczyk A, Hałubiec P, Szafrański O, Karnas K, Karewicz A. Superparamagnetic iron oxide nanoparticles-current and prospective medical applications. Materials (Basel). 2019;12(4). 10.3390/ma12040617 PMC6416629 30791358

[49] Wabler M, Zhu W, Hedayati M, Attaluri A, Zhou H, Mihalic J (2014). Magnetic resonance imaging contrast of iron oxide nanoparticles developed for hyperthermia is dominated by iron content. Int J Hyperth.

[50] Rybka JD (2019). Radiosensitizing properties of magnetic hyperthermia mediated by superparamagnetic iron oxide nanoparticles (SPIONs) on human cutaneous melanoma cell lines. Rep Pract Oncol Radiother.

[51] Zheng SW, Huang M, Hong RY, Deng SM, Cheng LF, Gao B (2014). RGD-conjugated iron oxide magnetic nanoparticles for magnetic resonance imaging contrast enhancement and hyperthermia. J Biomater Appl.

[52] Kandasamy G, Sudame A, Luthra T, Saini K, Maity D (2018). Functionalized hydrophilic superparamagnetic iron oxide nanoparticles for magnetic fluid hyperthermia application in liver cancer treatment. ACS Omega.

[53] Na HB, Song IC, Hyeon T (2009). Inorganic nanoparticles for MRI contrast agents. Adv Mater.

[54] Hu P, Fu Z, Liu G, Tan H, Xiao J, Shie H, et al. Gadolinium-based nanoparticles for theranostic MRI-guided radiosensitization in hepatocellular carcinoma. Front Bioeng Biotechnol. 2019. 10.3389/fbioe.2019.00368 PMC6890599 31828068

[55] Klein S, Sommer A, Distel LVR, Neuhuber W, Kryschi C (2012). Superparamagnetic iron oxide nanoparticles as radiosensitizer via enhanced reactive oxygen species formation. Biochem Biophys Res Commun.

[56] Kirakli E, Takan G, Hoca S, Muftuler FZ (2018). Superparamagnetic iron oxide nanoparticle ( SPION ) mediated in vitro radiosensitization at megavoltage radiation energies. J Radioanal Nucl Chem.

[57] Seo S-J, Jeon J-K, Jeong E-J, Chang W-S, Choi G-H, Kim J-K. Enhancement of tumour regression by coulomb nanoradiator effect in proton treatmetn of iron-oxide nanoparticle-loaded orthotopic rat glioma model: implication of novel particle induced radiation therapy. J Cancer Ther. 2013;4(11A).

[58] Choi G-H, Seo S-J, Kim K-H, Kim H-T, Park S-H, Lim J-H, et al. Photon activated therapy (PAT) using monochromatic synchrotron x-rays and iron oxide nanoparticles in a mouse tumor model: feasibility study of PAT for the treatment of superficial malignancy. Radiat Oncol. 2012;7(182). 10.1186/1748-717X-7-184 PMC3549855 23111059

[59] Andreo P, Burns DT, Nahum AE, Seuntjens J, Attix FH. Fundamentals of Ionizing Radiation Dosimetry. WIley; 2017.

[60] Hubbell JH, Seltzer SM. Tables of X-ray mass attenuation coefficients and mass energy-absorption coefficients 1 keV to 20 MeV for elements Z= 1 to 92 and 48 additional substances of dosimetric interest (No. PB-95–220539/XAV; NISTIR-5632). Gaithersburg, MD (United States); 1995.

[61] Iron Oxide Nanoparticles Product specification [Internet]. Sigma-Aldrich. Available from: https://api.sigmaaldrich.com/deepweb/assets/sigmaaldrich/quality/spec/211/319/725331-BULK-ALDRICH.pdf.

[62] Minelli C, Bartczak D, Peters R, Rissler J, Undas A, Sikora A (2019). Sticky measurement problem: number concentration of agglomerated nanoparticles. Langmuir.

[63] Russell B, Garcia-Miranda M, Ivanov P (2017). Development of an optimised method for analysis of 90Sr in decommissioning of wastes by triple quadrupole inductively coupled plasma mass spectrometry. Appl Radiat Isot.

[64] Taylor R (1990). Interpretation of the Correlation coefficient: a basic review. J Diagn Med Sonogr.

[65] Puck T, Marcus P (1956). Action of X-rays on mammalian cells. J Exp Med.

[66] Joiner M, Van Der Kogel A. Basic clinical radiobiology. 4th ed. Hodder Arnold; 2009. 375 p.

[67] Khoshgard K, Kiani P, Haghparast A, Hesseinzadeh L, Eivazi M. Radiation dose rate affects the radiosensitization of MCF-7 and HeLa cell lines to X-rays induced by dextran-coated iron oxide nanoparticles. Int J Radiat Biol. 2017;93(8). 10.1080/09553002.2017.1321806 28452253

[68] Russell E, Mcmahon SJ, Russell B, Mohamud H, Mcgarry CK, Schettino G (2020). Effects of gadolinium MRI contrast agents on DNA damage and cell survival when used in combination with radiation. Radiat Res.

[69] Ahmad R, Schettino G, Royle G, Barry M, Pankhurst QA, Tillement O, et al. Radiobiological implications of nanoparticles following radiation treatment. Part Part Syst Charact 2020;37(4). 10.1002/ppsc.201900411 PMC8427468 34526737

[70] Kehrer JP (2000). The Haber–Weiss reaction and mechanisms of toxicity. Toxicology.

[71] Dufort S, Le Duc G, Salomé M, Bentivegna V, Sancey L, Bräuer-Krisch E (2016). The high radiosensitizing efficiency of a trace of gadolinium-based nanoparticles in tumors. Nat Sci Rep.

[72] Lanier OL, Korotych OI, Monsalve AG, Wable D, Savliwala S, Grooms NWF (2019). Evaluation of magnetic nanoparticles for magnetic fluid hyperthermia. Int J Hyperth.

[73] Gutiérrez L, De La Cueva L, Moros M, Mazarío E, De Bernardo S, De La Fuente JM, et al. Aggregation effects on the magnetic properties of iron oxide colloids. Nanotechnology. 2019;30(11). 10.1088/1361-6528/aafbff 30609414

[74] Ahsan SM, Rao CM, Ahmad MF (2018). Nanoparticle-protein interaction: the significance and role of protein corona. Adv Exp Med Biol.

