# Supplementary material for: Impact of superparamagnetic iron oxide nanoparticles on in vitro and in vivo radiosensitisation of cancer cells
Source: Radiat Oncol. 2021 Jun 12;16:104. doi: 10.1186/s13014-021-01829-y (PMC8199842; doi:10.1186/s13014-021-01829-y)
Supplement: Supplementary file 1 — Additional file 1. Graph of time against correlation coefficient for 3 repeat measurements. It is a near perfect sigmoidal curve starting at 0.9 on the y-axis suggesting no aggregation of nanoparticles. [file 13014_2021_1829_MOESM1_ESM.pdf]

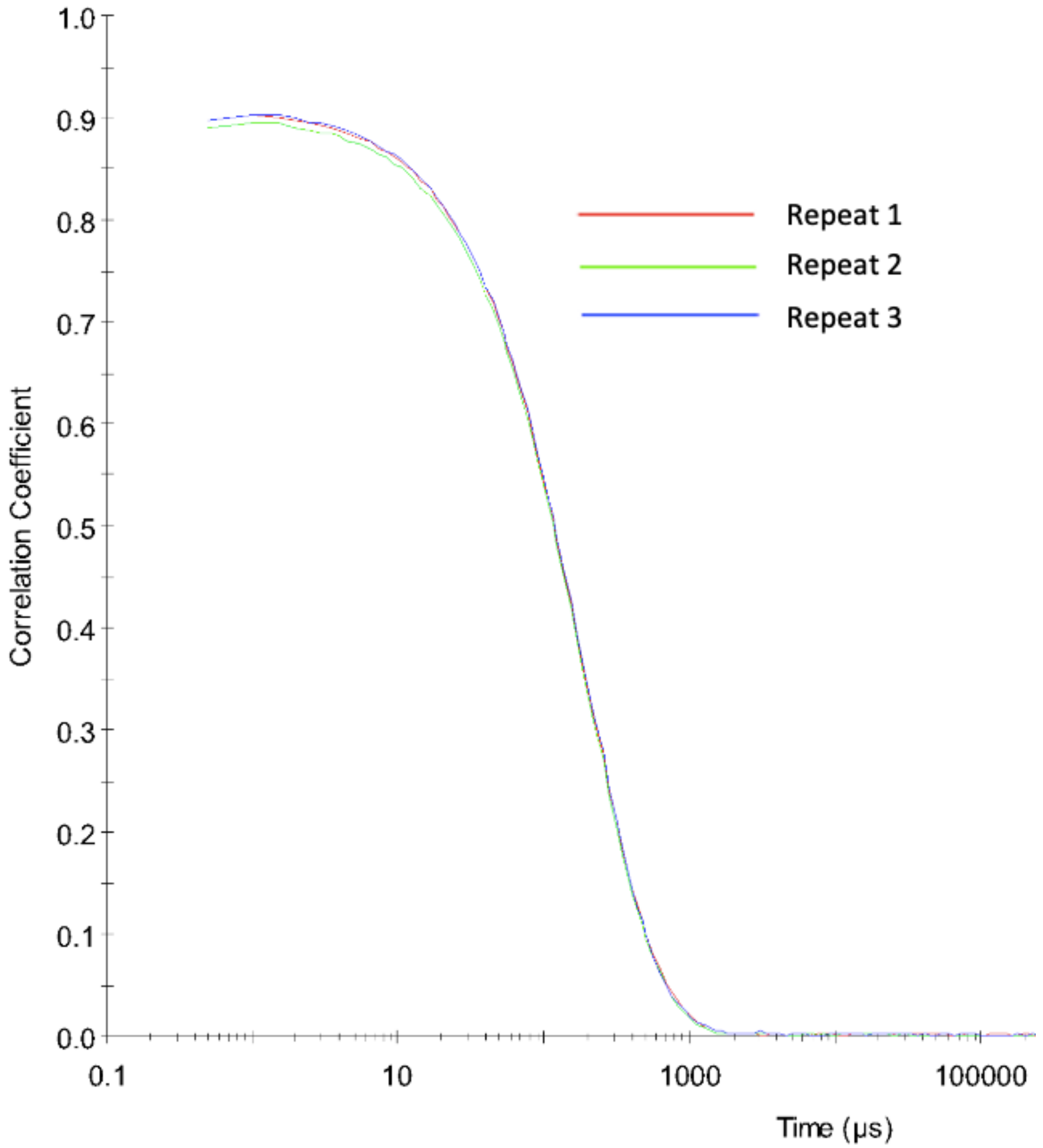

Appendix 1
Graph of time against correlation coefficient for 3 repeat measurements. It is a near perfect sigmoidal curve starting at 0.9 on the y-axis suggesting no aggregation of nanoparticles.
