# Supplementary figures and images for: Impact of superparamagnetic iron oxide nanoparticles on in vitro and in vivo radiosensitisation of cancer cells
Source: Radiat Oncol. 2021 Jun 12;16:104. doi: 10.1186/s13014-021-01829-y (PMC8199842; doi:10.1186/s13014-021-01829-y)

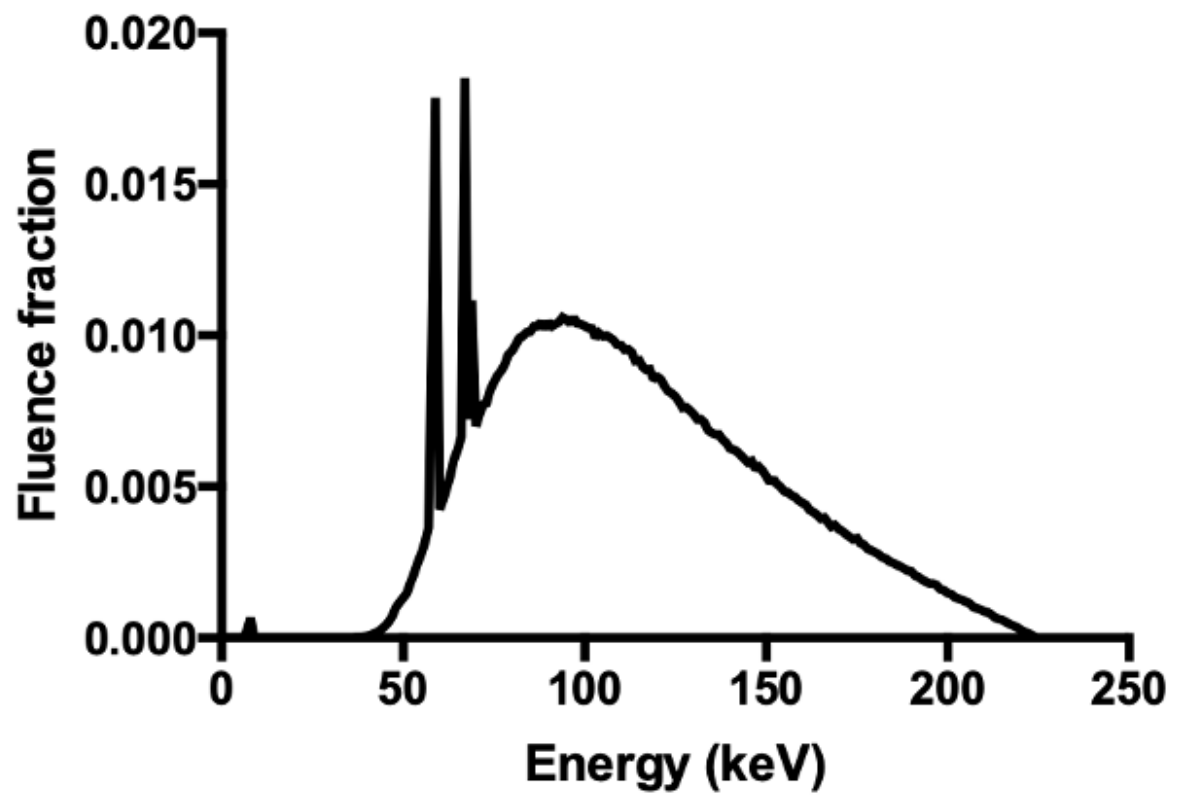

#### Appendix 2

Graph of the filtered x-ray beam spectrum used for the *in vitro* experimentation.

Supplement: Supplementary file 2 — Additional file 2. Graph of the filtered x-ray spectrum used for the in vitro experimentation. [file 13014_2021_1829_MOESM2_ESM.pdf]
