# Supplementary material for: Impact of superparamagnetic iron oxide nanoparticles on in vitro and in vivo radiosensitisation of cancer cells
Source: Radiat Oncol. 2021 Jun 12;16:104. doi: 10.1186/s13014-021-01829-y (PMC8199842; doi:10.1186/s13014-021-01829-y)
Supplement: Supplementary file 3 — Additional file 3. Graph of energy against DEF for the range of energies in the x-ray spectrum used for in vitro experimentation. [file 13014_2021_1829_MOESM3_ESM.pdf]

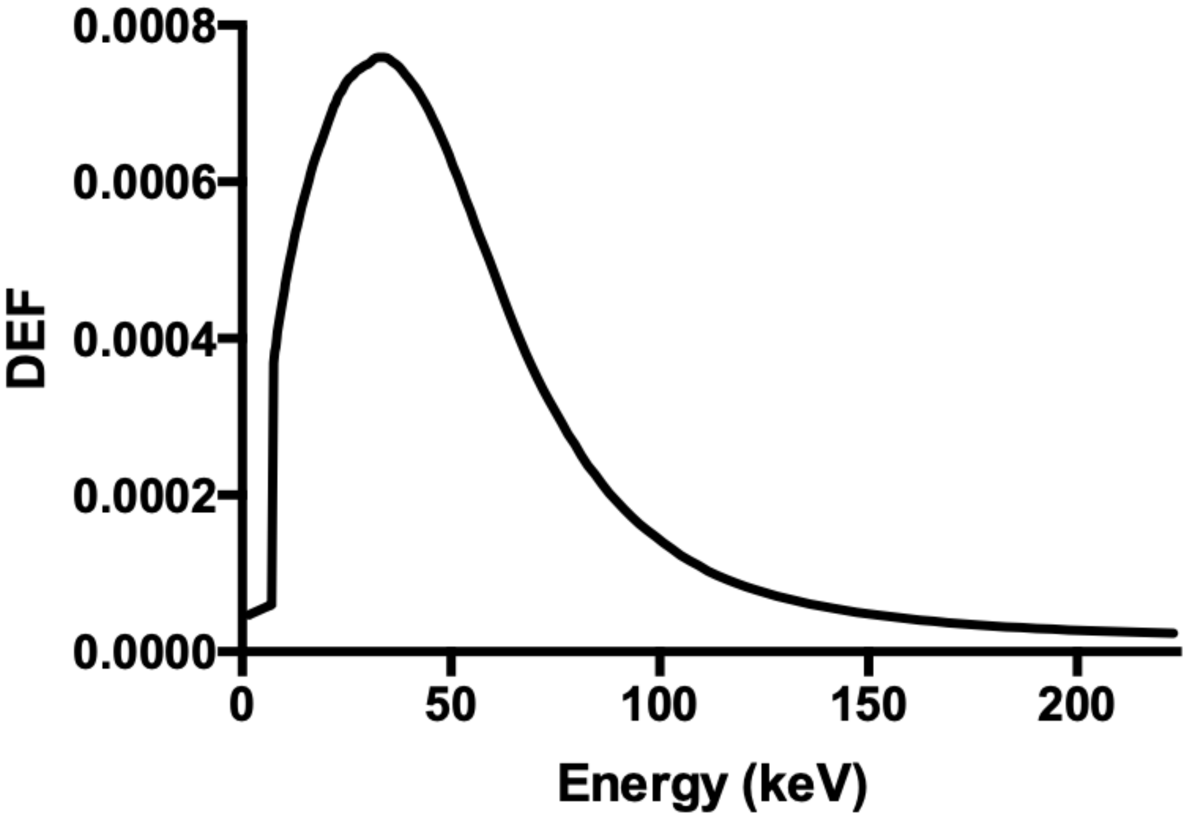

Appendix 3
Graph of energy against DEF for the range of energies in the x-ray spectrum used for *in vitro* experimentation.
